# The Gender Wage Gap and Parenthood: Occupational Characteristics Across European Countries

**DOI:** 10.1007/s10680-023-09681-4

**Published:** 2023-11-30

**Authors:** Alícia Adsera, Federica Querin

**Affiliations:** 1 A29 JRR Building, Princeton University, Princeton, NJ 08544 USA; 2 University of Bologna, Bologna, Italy

**Keywords:** Gender wage gap, ONET characteristics, Motherhood penalty, Leadership, Autonomy

## Abstract

Different strands of research analyse gender occupational differences and how they relate to differential earnings, especially among parents juggling family demands. We use rich data from PIAAC across a subset of European countries and match occupational characteristics to individuals’ jobs using the O*NET database to analyse, first, whether there are gender differences in the occupational characteristics of jobs, particularly among parents, and second, whether the return to key occupational characteristics varies by gender. Compared to men, women’s jobs generally require more contact with others, less autonomy in decision-making, and less time pressure. In addition, positions held by mothers involve both less leadership expectations and less intensive use of machines than those held by fathers. Further, mothers receive a lower return to both of these occupational characteristics than fathers do. Finally, even though gaps in occupational characteristics such as leadership jointly with the differential sorting of mothers and fathers across sectors explain part of the gender wage gap in Oaxaca–Blinder decomposition models, especially in Continental Europe, a large share remains unexplained particularly in Eastern and Southern European countries.

## Introduction

Despite important advances in closing the gender wage gap in most advanced nations during the last decades, progress has slowed down or stalled during recent years (England, 2010). Different strands of research suggest mechanisms to explain the observed gender wage gap and how it relates to differences in occupational characteristics and work effort (Blau & Kahn, 2017). The need of families to accommodate (sometimes conflicting) work and family demands, particularly around childbearing, has long been a prominent subject in this work. Within couples, women (especially mothers) tend work fewer hours, select into education majors (Charles & Bradley, 2009) and, subsequently, into occupations with characteristics that more easily accommodate those needs. In the context of large labour market structural changes, with a concurrent increase in inequality and the loss of many middle-paying jobs among those women considered more friendly, this strategy can lead to increasing inequality within and across families and affect family formation and fertility choices (Adserà, 2017; Autor et al., 2003).

Women’s educational advances, often surpassing men’s, have contributed to the closing of the gender wage gap (Blau & Kahn, 2017). However, mothers still face a penalty compared to fathers due to career interruptions as confirmed by recent data in both the USA and Europe (Killewald & Gough, 2013; Kleven et al., 2019a, 2022). Once demographic characteristics are accounted for, the persistence of gender and parenthood gaps is often attributed to sorting into different sectors (Blau & Kahn, 2017) and into occupational characteristics within the sector (De La Rica et al., 2020; Felfe, 2012; Yu & Kuo, 2017).

In this paper, we study to what extent there is sorting on occupational characteristics and its implications for earnings differentials by gender, with a focus on parents. To this aim, we use rich data from the Program for the International Assessment of Adult Competencies (PIAAC) for a sample of fourteen countries and match occupational characteristics at 4-digit level to individuals’ jobs using O*NET database. In particular, we combine several occupational characteristics into five factors (contact with others, leadership, autonomy, machine dependency, and time pressure) as we detail below and analyse whether their prevalence as well as their wage returns vary by gender and by parenthood.

We first focus on occupational characteristics that have an interpersonal component. Because of different expected wage returns, we separate occupations that entail contact with peers such as colleagues, clients, and the public (contact with others) from those that are hierarchical in nature and give rise to better-paid leadership positions (leadership). We then turn to occupational characteristics that afford the worker autonomy in decision-making (autonomy) and others that instead imply dependency on heavy machinery, where men are likely overrepresented but do not afford high returns (machine dependency). Lastly, we assess the prevalence and returns of the time pressure exerted by frequent deadlines by gender and separately for parents (with children of different ages) who may be under tighter time constraints, especially if their children are younger (time pressure). As we detail below, European countries differ in welfare state provisions, gender norms, and labour market structures. We expect underlying country-specific conditions in these dimensions to impact the extent of gender differences in the prevalence of occupational characteristics as well as gender wage gaps. Therefore, besides pooled country estimates, we provide estimates separate by countries grouped according to industrial relations classification by Eurofound (2020).

The empirical analysis of the paper contributes to the understanding of gendered nature of work and wage returns for parents (compared to the general population) by addressing three questions. The first set of empirical analyses documents profound differences in prevalence, especially for contact with others (predominantly female) and machine dependency (predominantly male). This is accentuated in Continental Europe and for parents. The second set of estimates analyses the wage returns to those occupational characteristics and whether they differ by gender, parenthood, and country groups. The gender wage gap is largest for parents of young children and in Southern and Eastern Europe. Higher wage returns in occupations with high autonomy and leadership increase the gender wage gap, but lower pay in machine-dependent occupations partially closes it. Findings from the last set of analyses, Oaxaca–Blinder decomposition of the gender gap, are consistent with those on prevalence and return. A final discussion section concludes the paper.

## Gender, Parenthood and Occupational Changes

### The Gender Wage Gap and Occupational Sorting

After important progress since the 1960s, the closing of the gender wage gap has slowed down or stalled in recent years (England, 2010). While differences in wages were previously explained to a large degree by measurable characteristics such as differences in human capital across gender (i.e. education, training, work experience), the steep increase in women’s educational success has mostly eliminated that source of wage differentials (Blau & Kahn, 2017). However, differences in experience still persist, in part due to a more intermittent attachment of women to the labour force in both the intensive and extensive margins (Denning et al., 2019; Felfe, 2012; Hirsch, 2005; Kleven et al., 2019b, 2022; Lucifora et al., 2021; Mas & Pallais, 2020). Even after controlling for work effort and education, recent analyses via Oaxaca–Blinder decomposition techniques suggest that sectoral and occupational differences are key to explaining the remaining gender gap (see Blau & Kahn, 2017 for US data; Leythienne & Ronkowski, 2018 for European data). Selection across different sectors or occupations can result from explicit choices made by individuals or alternatively from other forces such as discrimination or barriers to entry (insider/outsider dual market, for example). Further, differences in job characteristics related, for example, to the intensity of required presence at work, or differences in workers’ competitiveness may explain part of the remaining wage gap (Gneezy et al., 2003). Goldin (2014) shows that job features such as time pressure, unstructured work and the importance of maintaining personal relationships, among others, are associated with nonlinear hourly wage profiles by effort among highly educated workers and that gender earnings differences are larger in sectors intensive on those characteristics.

Complementing these mechanisms, Levanon and Grusky (2016) argue that the persistence of occupational segregation, and accompanying wage differences, is due to two distinct cultural principles that are interwoven to generate the observed patterns. First, the essentialist presumption invokes the existence of fundamentally different tastes by gender that result in different occupational choices. Second, wages tend to be higher in tasks in which men are believed to have comparative advantages and in male-dominated fields (Levanon & Grusky, 2016).

### The Motherhood Penalty

Gendered occupational sorting increases in saliency for parents (Killewald & Gough, 2013). Previous work on family formation considers specialization within couples as an important driver of gender differences in employment and wages. First, even though there is some ongoing debate about the extent of specialization within different family arrangements, most research notes that women entering marriage tend to decrease labour market effort, and they do so more than women entering more unstable cohabitations, while men increase it (Adserà & Querin, 2021; Ginther et al., 2010; Kalenkoski et al., 2005; South & Spitze, 1994; Stratton, 2002). The drop in young married women’s work is especially large in contexts where men work long hours (Cortes & Pan, 2017).

Second, the literature on fertility points at the conflict between family and work resulting in late (and less) childbearing when women either face uncertainty in the labour market or aim to achieve career goals ahead of motherhood (Adserà, 2005, 2011a, 2011b; Alderotti et al., 2021). In addition, this conflict likely implies large drops in labour market in both the intensive and the extensive margin of women around childbirth, as well as in the following years, that may be lessened by the institutional support offered to mothers (Gornick & Meyers, 2003; Mandel & Semyonov, 2006; McDonald, 2000; Olivetti & Petrongolo, 2017; Thévenon, 2011). Thus, there is a general consensus on the existence of a motherhood penalty, suggesting that intra-household specialization, changes in hours worked, and type of job become more salient with parenthood (Cukrowska-Torzewska & Matysiak, 2020; Glauber, 2007; Killewald & Gough, 2013; Kleven et al., 2019b, 2022). For example, the number of children has regularly been found to account for a substantial part of earnings differences across women, but not that much across men (Lundberg & Rose, 2000; Waldfogel, 1997, 1998). Mothers tend to work fewer hours and select into occupations with characteristics that more easily accommodate those needs, but that often command lower wages (Charles & Bradley, 2009; Felfe, 2012; Kleven et al., 2019a, 2019b). This is in line with compensating differentials theory, which proposes that workers are willing to take lower wages in exchange for desirable job characteristics (Rosen, 1986), in the specific case of women for jobs compatible with parenting (Filer, 1985). For example, part-time jobs and positions in the public sector securing employment continuity and generous leaves are associated with faster transitions to births in Europe and continuous labour market attachment of mothers, despite lower wages (Adserà, 2011a, 2011b). Lucifora et al. (2021) show that the gender gap in France widens around childbirth as mothers may miss promotions by not being present at work so intensively. Further, new mothers may relocate closer to home and trade-off more rewarding job opportunities for this amenity as low commuting cost is high in the list of job amenities mothers value (Petrongolo & Ronchi, 2020).

### Occupational Characteristics

Occupational and sectoral sorting remain important elements contributing to the persistence of the gender and parenthood wage gap potentially for two reasons (Blau & Kahn, 2017): first, if the prevalence of occupational characteristics differs across men, women, mothers, and fathers significantly, and/or second, if wage returns to such characteristics vary by gender and parenthood status. Building on the body of works below, we introduce occupational characteristics that could contribute to explain the gender and parenthood wage gaps beyond sectoral differences.

The first is *contact with others*, which has been at centre stage of the broader debate on flexibility and the ability to work from home, a literature that has assumed particular prominence during the COVID-19 pandemic (Albanesi & Kim, 2021; Barbieri et al., 2020; Bonacini et al., 2021; Gariety & Shaffer, 2007). While this body of work focuses primarily on those occupations that require face-to-face contact, transformations in the labour market brought about by advances in IT extend the realm of interpersonal working relationships beyond it (Koren & Pető, 2020). For example, Goldin (2014) conceptualizes contact with others and establishing interpersonal relationships regardless of mode of contact, but her focus is limited to highly educated workers. Working in contact with others, be it within a team or with external customers and/or the public, requires what Levanon and Grusky (2016) describe as “sociability,” an attribute associated more with women than men (Cejka & Eagly, 1999; Levanon & Grusky, 2016). Often these occupations are associated with lower wages, which in turn increases the gender wage gap when women are overrepresented in them (Charles & Bradley, 2009; Gorman, 2005). Therefore, we hypothesize *contact* with others will be more prevalent in jobs held by women than men—maybe slightly more moderately among mothers who may worry about the dependency of their presence requirements—but that returns to this occupational characteristic will not be high.

While our conceptualization of contact with others pertains to clients, team members, and colleagues, jobs often entail a hierarchical structure. The different roles within such structures give rise to interpersonal relationships across “vertical lines.” *Leadership* roles, often managerial in nature, involve the guiding, directing, and coordinating of other workers. Despite the fact that these activities could be associated with “sociability,” they are often gendered male (Levanon & Grusky, 2016). The large literature on the existence and extent of a glass ceiling effect for women in leadership positions also highlights the higher wage returns men reap from them (Arulampalam et al., 2007; Mandel & Semyonov, 2006). Therefore, we hypothesize that men’s jobs will have a higher prevalence of leadership and receive relatively higher wage returns to them, especially fathers.

*Autonomy* can be defined as having a job characterized by control on the decision-making process (Fielding, 1990) and on how to structure the work to be done. Freedom to make decisions and the frequency with which a worker does it are often, but not only, associated with managerial positions (Wheatley, 2017) and therefore expected to be correlated with leadership and higher wages (Goldin, 2014; Green, 2007). However, we argue that autonomy is separate from leadership insofar as it does not explicitly entail contact with others to the same extent and does not necessarily require providing guidance or direction to other employees. On the same grounds, even though we expect autonomy to be correlated with some extent with contact, there are many jobs that do not require direct continuous contact with others, but offer high flexibility in decisions and work structure (i.e. text editing), while the reverse is also true (i.e. post office employee).

On the one hand, occupations characterized by more autonomy could be more attractive to women and parents who can retain more control over their workflow and more easily juggle with domestic demands. On the other hand, more decision-making is often associated with extensive and unpredictable work time because of consequential impact of decisions on co-workers or company results (Wheatley, 2007), therefore making those positions with more autonomy less attractive to women and parents. When balancing these two opposing forces, we hypothesize the prevalence of autonomy in jobs across gender to be similar. While we expect autonomy to bear high wages (Green, 2007), predictions for gender differentials in wage returns to autonomy are ambiguous given that women and men should in theory be equally rewarded for holding decision-making power.

On the opposite spectrum of autonomy, there are jobs that are considered highly inflexible because of the need to directly use machinery and operating vehicles that demand both the physical presence of the worker at the workplace and a work structure constrained by the tools of the job (Albanesi & Kim, 2021; Mas & Pallais, 2020). These *machine-dependent*^1^ occupations are often heavily skewed towards men (Levanon & Grusky, 2016) and, unlike most others predominantly male occupations, do not necessarily reap high wage returns. Therefore, we hypothesize that machine dependence will be more prevalent in men’s jobs (without a clear association with parenthood) and that this will penalize men in terms of wages.

An element that cuts across all these occupational characteristics is time. Arguably, working long and/or odd hours and having *time pressure* (deadlines) likely conflicts with family needs (Edin & Shaefer, 2015). However, compensating differentials may apply to jobs with frequent deadlines and associated fluctuations in the workload. For example, in a survey among workers in a calling centre, Mas and Pallais (2017) found that workers are in fact willing to suffer up to a 20% reduction in their wages to avoid unexpected shifts in their schedule. Time pressure can make having continuous deadlines less desirable for mothers who are often working a double shift, although there may not be gender differences for childless individuals. This might be especially relevant in contexts in which men do not evenly share household tasks (de Laat & Sevilla-Sanz, 2011). Therefore, we hypothesize a lower prevalence of time pressure for mothers (but ambiguous for childless individuals) and likely higher wage returns.

### Cross-Country European Contexts

Countries in the PIAAC sample vary along dimensions likely important to interpret findings on occupational sorting and motherhood penalty. Our analyses group countries according to the classification used by Eurofound (2020) that captures industrial relations in the EU and that nicely aligns with literature that focuses on differences in welfare systems and gender norms across Europe. These groups include Continental Europe (Belgium, Germany, France, the Netherlands), Southern Europe (Spain, Greece, Italy), Eastern Europe (Czech Republic, Lithuania, Poland, Slovenia, Slovakia), the UK to represent Anglo-Saxon and Denmark to represent Nordic countries.

In terms of recent sectoral employment changes, both Anglo-Saxon and Nordic countries have experienced the largest decreases in industrial employment and transportation and these sectors have modernized, become more automatized, and are probably more welcoming to women on the factory floor. Conversely, those sectors have been relatively resilient, or grown in the case of transportation, in Eastern Europe. In the UK and Denmark, service sectors, ranging from finance to those typically associated with women such as education and health, have expanded (Eurofound, 2020). The weight of services in Continental and Southern European countries has also increased, but at a more moderate pace. In Southern Europe growth is concentrated in services related to commerce and hospitality which tend to employ women and young workers, but do not offer high wages. Thus, the expansion of the service sector in these countries is not expected to contribute as much to the closing of the gender wage gap.

The structure of the labour relations impacts the careers of workers and mothers in particular. Labour market institutions in the UK are more liberal, but flexible to accommodate different degrees of attachment. Conversely, in Southern Europe, protection for permanent workers hurts relatively women and young workers who are overrepresented in temporary work and unemployment (Adserà, 2005, 2011a). Sizable and stable public sector employment allows women to more easily enter and stay attached to the labour force in Nordic countries.

Family policies vary widely across Europe, starting with initiatives to support fertility and general family programmes (Thévenon, 2011). Family expenditure ranges from the lowest shares of GDP in Greece and Spain around 1–1.3% and the highest in Denmark, France and the UK around 3.4–3.7% according to 2019 Eurostat data. The extent to which countries offer family reconciliation policies that help mothers to remain attached to the labour force also varies widely across Europe. Shares of coverage are the lowest in Southern Europe, where average firm size is small and subsidies from the public sector weak (Eurostat 2019). Parental leave supports mothers’ attachment to the workforce, but could foster occupational sorting in which women gravitate (or else are channelled) to positions where they can be more easily replaced when absent or with less autonomy and leadership expectations (Janta, 2014; Mandel & Semyonov, 2006; Olivetti & Petrongolo, 2017), thus exacerbating the gender wage gap. Similarly, childcare availability is high in Nordic countries and France, and low in Germany, and in most Eastern Europe (Szelewa & Polakowski, 2008). While some of these policies have been successful into sustaining labour force participation of mothers, some argue that they have not always been accompanied with increases in gender occupational equality and a closing of the gender wage gap (Gornick & Meyers, 2003; Mandel & Semyonov, 2006) while others find moderate positive gains (Olivetti & Petrongolo, 2017).

Finally, researchers have long pointed to the need of a gender revolution to ultimately revert falling fertility rates while allowing mothers to remain attached to the market (McDonald, 2000). European countries still display large heterogeneity in gender norms regarding intra-household specialization and sharing of household work, with very poor indicators in Germany or Southern Europe compared to Nordic countries (Angelov et al., 2016; de Laat & Sevilla-Sanz, 2011). All these sectoral, institutional and policy differences combined with social norms around the work of mothers give rise to a large heterogeneity in the motherhood penalty in Europe (Cukrowska-Torzewska & Matysiak, 2020; de Linde Leonard & Stanley, 2020; Kleven et al., 2019a, 2019b, 2022; Leythienne & Ronkowski, 2018; Redmond & Mcguinness, 2019).

## Data and Methods

### Occupational Characteristics: O*NET Factors

For each currently employed individual, we match occupational characteristics at 4-digit ISCO-08 codes with information retrieved from the O*NET database. The database, available online https://www.onetonline.org/, has detailed descriptions of the requirements and work content and characteristics of over 900 occupations in the USA.^2^

While Table 8 in Appendix provides an item-by-item description of the construction of the O*NET factors used in the analyses as well as the factor loadings of each one of the components, here we briefly describe their main characterizations. All factors are normalized to have mean of zero and a standard deviation of one. First, *contact with others* captures the importance of communicating with others (be it colleagues, external customers, or the public) without a clear hierarchical structure in the relationship. It reflects and extends factors presented in Goldin (2014) who suggests more contact means less flexibility as others need the worker to be present in order to carry out her job. Second, our measure of *leadership* also reflects strong interpersonal contact with others. However, unlike contact with others, items in this factor explicitly refer to relationships that are hierarchical in nature and where the worker has a coordinating and guiding role (e.g. coordinating the work and activities of others, guiding, directing, and motivating subordinates, etc.). Therefore, there is still a need for interpersonal relationships to fulfil one’s job, but the worker has more latitude in directing them. Third, *autonomy* relates to the importance, frequency, and freedom to take up the role of decision-maker. While this measure could be correlated with both leadership and contact with others, it is distinct from the former by the absence of an explicit relationship with others and from the latter as workers enjoy a large degree of autonomy in some occupations that do not require extensive interaction with other co-workers. Fourth, *machine dependency* is built by selecting, from the measures of inflexibility suggested in the literature, the items pertaining specifically to the operation of mechanized devices, moving objects, and the control of machines and processes (see for example Albanesi & Kim, 2021). This factor explicitly excludes computers as machines and therefore captures features of blue-collar occupations more than the other factors. Lastly, *time pressure* captures the time sensitivity of the job, measured by how often the job requires the worker to meet strict deadlines, ranging from never to every day. Correlations across factors are reported in Appendix Table 9.

As similar comparable data across our countries is limited, we consider appropriate to use the O*NET database even though it is based on US occupations for two reasons. First, it affords a very rich occupational classification to construct different types of requirements and, second, an extensive literature has already employed it jointly with different European datasets. The implicit assumption in all cases is that the task content of occupations in the USA and in European countries, including CEE countries, is similar in relation to the measured skill dimensions (Handel, 2012; Hardy et al., 2018), D’Amuri and Peri (2014) use it to construct an index of occupational complexity and merge it with the EU-LFS. Ortega and Polavieja (2012) construct indexes for manual and communication skills in each occupation and combine them with the European Social Survey. Amuedo-Dorantes and De la Rica (2011) use the O*NET to check whether there is a correlation between changes in the employment shares by occupations and task intensities in the Spanish labour market. Cedefop (2013) shows that results from two surveys based on O*NET conducted in Italy and Czech correlated highly with those of O*NET. Finally, De la Rica et al. (2020) do the same with the PIAAC data employed in this paper.

### Individual-Level Data

The study employs data from the Program for the International Assessment of Adult Competencies (PIAAC), also known as the Survey of Adult Skills, This is a multi-country harmonized dataset collected by the OECD starting in 2012 with the aim to measure key cognitive and workplace skills (https://www.oecd.org/skills/piaac/). Our main analytic sample includes men and women between 16 and 65 years of age who were wage and salary workers in all sectors including agriculture. We focus on those who worked at least 20 h a week in their current job to identify workers who have substantial level of commitment to the labour force. This leads to a sample of 39,709 in 14 countries: Belgium, Czechia, Germany, Denmark, Spain, France, Greece, Italy, Lithuania, the Netherlands, Poland, Slovenia, Slovak Republic, and UK. Conscious of gender and country differences in the prevalence of part-time work, we conducted further analyses relaxing the minimum hour restriction and results were similar to those shown here. Before any restrictions, the sample is similarly divided between men and women, but men are more likely to be childless than women in this sample, leading to a sizeable group of mothers. Adding the work requirement restrictions reduces the sample size gap between mothers and fathers, but does not completely close it. Therefore, the higher number of working mothers compared to fathers is not the result of higher employment rates of mothers, but rather of higher rates of motherhood than fatherhood in the sample.

### Methods

We conduct our analyses along the three main questions of interest. The first set of models tests the existence of differences in the prevalence of our occupational characteristics by gender and separately by parenthood status, both for the whole sample and across of groups of countries. The dependent variable in these models is the occupational characteristic of interest and the key independent variable is gender. Additionally, analyses among parents are stratified by the age of the youngest child.

The second set of models analyses differences in the logarithm of hourly wages^3^ by gender and controls for the five occupational characteristics both in levels and interacted by gender to determine whether the return to those characteristics varies by gender and parenthood status.

The third and last set of analyses uses an Oaxaca–Blinder decomposition to investigate whether the differential sorting of fathers and mothers across jobs contributes to explain wage differences. We let β_m_ and β_f_ be, respectively, the vector of OLS estimates of a Mincer log wage equation for men *Y*_m_ and for women *Y*_f_, respectively, and denote mean values of the vector of relevant characteristics $\overline{X}$. In addition, we obtain *β*_p_ the vector of OLS estimates from a pooled model that also includes an indicator for gender (Neumark, 1998). Because OLS with a constant term produces residuals with a zero mean, we can write the difference in log average wages by gender as follows:

$$ \overline{{Y_{\text{m}} }} - \overline{{Y_{\text{f}} }} = \beta_{\text{p}} (\overline{{X_{\text{m}} }} - \overline{{X_{\text{f}} }}) + \overline{{X_{\text{m}} }}(\beta_{\text{m}} - \beta_{\text{p}} ) + \overline{{X_{\text{f}} }}(\beta_{\text{p}} - \beta_{\text{f}} ) $$

The first term is the part of the gender wage gap “explained” by group differences in the variables; it evaluates the gender differences in characteristics using the coefficients from the pooled wage equation *β*_p_. The second part is the unexplained differential which can include both discrimination and differences in unobserved variables. In the tables, we show the part of the gap explained by each of the components of our models.

Unless otherwise specified, all models in the paper include country fixed effects, age, age squared, and whether the respondent completed secondary or tertiary education. In addition, models control for whether the position held by the worker is temporary, within the public sector and its 2-digit ISIC sector (Blau & Kahn, 2017).

Due to PIAAC complex sampling strategy, we present weighted estimates to compensate for the disproportionate sampling of subgroups and non-coverage. Therefore, following PIAAC guidelines, main results are presented with paired jackknife standard errors (PIAAC Technical report, 2019).^4^

## Descriptive Results

Table 1 reports descriptive statistics for both women and men and by parenthood status. Consistent with recent aggregate data across European countries, women have a higher educational attainment than men in the sample. The level of education is particularly high among mothers with a child under 12, as 32% of them have tertiary education. Given that our sample requires them to be working more than 20 h a week, workers may be somewhat positively selected. Men’s log hourly wage is higher than women’s and the gender difference is somewhat larger among parents (especially those with children under 12). Fathers work longer hours (around 42 h) than mothers (around 35 h) per week. With regard to the type of job, the share of parents in a temporary contract is lower than the average, hinting to some employment stability before/at the time parenthood (especially for fathers). Women are overrepresented in the public sector with around a third of them holding this type of job.

**Table 1:** Descriptive statistics

	(1) All women	(2) All men	(3) Mothers	(4) Fathers	(5) Mothers child under 12	(6) Fathers child under 12
Contact	0.173 (0.985)	− 0.206 (0.954)	0.117 (0.999)	− 0.185 (0.996)	0.218 (0.932)	− 0.168 (0.958)
Autonomy	− 0.071 (1.003)	− 0.045 (0.886)	− 0.113 (1.008)	0.007 (0.904)	− 0.026 (0.985)	− 0.025 (0.856)
Time pressure	− 0.106 (1.131)	0.210 (0.848)	− 0.099 (1.085)	0.247 (0.838)	− 0.088 (1.087)	0.252 (0.799)
Leadership	− 0.143 (1.074)	0.015 (0.874)	− 0.142 (1.068)	0.077 (0.933)	− 0.080 (1.047)	0.087 (0.885)
Machine dependency	− 0.252 (0.794)	0.321 (0.957)	− 0.217 (0.777)	0.325 (0.987)	− 0.275 (0.771)	0.287 (0.967)
Log hourly Wage	2.628 (2.268)	2.774 (1.875)	2.640 (1.767)	2.859 (1.874)	2.553 (0.970)	2.817 (1.590)
Work hours	36.114 (8.808)	41.450 (7.282)	35.418 (8.692)	41.898 (7.273)	34.850 (8.459)	42.187 (6.990)
Temporary contract	0.153 (0.375)	0.142 (0.337)	0.125 (0.335)	0.104 (0.304)	0.159 (0.369)	0.120 (0.312)
Public sector	0.333 (0.490)	0.188 (0.377)	0.365 (0.487)	0.208 (0.403)	0.352 (0.482)	0.181 (0.371)
Secondary education	0.587 (0.513)	0.587 (0.475)	0.599 (0.495)	0.596 (0.487)	0.564 (0.500)	0.577 (0.475)
Tertiary education	0.271 (0.463)	0.213 (0.395)	0.244 (0.434)	0.209 (0.404)	0.322 (0.471)	0.239 (0.411)
Observations	17,891	18,573	12,304	11,392	5486	5902

Analytic sample only. Weighted means, standard deviation in parentheses. Parents defined as responding yes to: “Do you have a child?.” Last two columns include parents who have at least one child under the age of 12. For O*NET factors definitions see text, all standardized to have mean zero and standard deviation 1. Authors’ calculations from PIAAC dataset

On average women are in jobs that have more contact with others compared to men. This gap is similar among parents. The level of autonomy is relatively similar across gender (a bit lower for all mothers). However, women tend to occupy positions with less time pressure for meeting deadlines and with less leadership. In terms of leadership, mothers are similar to all women, but fathers’ occupations display a higher degree than those of other men. Machine dependency is negatively (positively) associated with women’s (men’s) occupations. These general differences among the occupational requirements by gender and parenthood are confirmed by our estimates in the prevalence models below.

### The Prevalence of Job Characteristics Across Gender and by Parenthood

Estimates in Table 2 explore whether occupational characteristics vary across jobs held by men and women, and among parents in particular, according to the expectations laid out in the theory section. The female coefficient indicates to what extent women’s jobs have more or less of those requirements. As expected, women’s occupations require more contact with others—even after controlling for whether they work for the public sector. Mothers’ occupations are associated with 0.11 standard deviations more contact than fathers, while this difference increases to 0.25 standard deviations among childless individuals (see Table 10). In the third row of Table 10, we show this difference between parents and childless individuals to be significant in tests conducted in a fully interacted model.

**Table 2:** Prevalence of occupational characteristics by parenthood status

	Contact		Autonomy		Time pressure		Leadership		Machine-dependent	
	(1) All	(2) Parents	(3) All	(4) Parents	(5) All	(6) Parents	(7) All	(8) Parents	(9) All	(10) Parents
Female	0.16***	0.11***	− 0.12***	− 0.18***	− 0.13***	− 0.14***	− 0.31***	− 0.36***	− 0.36***	− 0.36***
	(0.016)	(0.022)	(0.016)	(0.022)	(0.018)	(0.023)	(0.016)	(0.023)	(0.014)	(0.017)
Secondary education	0.36*** (0.021)	0.39*** (0.027)	0.35*** (0.026)	0.36*** (0.026)	0.10*** (0.021)	0.07** (0.026)	0.10*** (0.019)	0.12*** (0.022)	− 0.28*** (0.018)	− 0.30*** (0.021)
Tertiary education	0.76*** (0.024)	0.82*** (0.031)	0.76*** (0.025)	0.81*** (0.031)	0.23*** (0.024)	0.23*** (0.030)	0.66*** (0.025)	0.74*** (0.030)	− 0.90*** (0.020)	− 0.92*** (0.025)
Public sector	0.06***	0.06**	0.07***	0.04	0.04	0.01	0.16***	0.14***	0.09***	0.08***
	(0.017)	(0.024)	(0.021)	(0.028)	(0.025)	(0.036)	(0.024)	(0.032)	(0.018)	(0.020)
Temporary contract	− 0.13*** (0.015)	− 0.16*** (0.023)	− 0.17*** (0.018)	− 0.20*** (0.025)	− 0.10*** (0.024)	− 0.12*** (0.029)	− 0.08*** (0.018)	− 0.09*** (0.026)	0.09*** (0.015)	0.10*** (0.021)
Constant	− 0.55***	− 0.37*	− 0.59***	− 0.26	− 0.13	0.19	− 0.36***	− 0.01	0.54***	0.53***
	(0.115)	(0.215)	(0.133)	(0.162)	(0.130)	(0.202)	(0.102)	(0.189)	(0.117)	(0.199)
Observations	36,464	23,696	36,464	23,696	36,464	23,696	36,464	23,696	36,464	23,696
R-squared	0.355	0.353	0.230	0.236	0.186	0.185	0.228	0.238	0.418	0.408

Weighted analytic sample. All models additionally include controls for age, age squared, sector at the ISIC 2-digit level, and country fixed effects. Parents defined as responding yes to: “Do you have a child?.” Paired jackknife standard errors in parentheses ***p < 0.01; **p < 0.05; *p < 0.1

Mothers’ occupations are characterized by around 0.18 standard deviations less of autonomy than jobs held by fathers, while there is no significant gender difference among the childless (Table 10). On the contrary, machine dependency is heavily represented in men’s jobs, as expected. Both mothers and childless women display over a third of a standard deviation less machine dependency than their peers. For time pressure, the gap of mothers with respect to fathers is similar for the overall gender gap, around 0.14 less standard deviations.

Finally, while all women hold fewer leadership positions, mothers are particularly excluded from those jobs with 0.36 standard deviation less on average than fathers. As expected, fathers are those with the highest leadership demands. Even though the gap is smaller for non-mothers, their jobs still display around 0.20 standard deviation less leadership requirements compared to childless men (as shown in Table 10)—which points to the existence of a glass ceiling effect for women in certain types of occupations.^5^

Estimates for controls are concordant with expectations. Education is positively associated with all occupational characteristics except for machine dependency. The educational gradient is the flattest for time pressure. Public sector employment is positively associated with all occupational features except for time pressure and autonomy among parents. This observation, jointly with women being almost twice as likely to be employed in the public sector than men, seems consistent with public positions being friendlier for mothers. Finally, temporary contracts are negatively associated with all factors (especially among parents) except for machine dependency.

### The Prevalence of Job Characteristics Across Gender and by Parenthood: Country Differences

Table 3 presents the female coefficient of models similar to those in Table 2 separately by groups of countries to analyse whether some of these associations are stronger in some contexts than others. Even though results are generally consistent with findings in Table 2, gender differences for both parents and childless workers across all occupational requirements are more moderate in the UK and Denmark than in other country groups and not even significant in autonomy for both and time pressure also in Denmark. The gap in machine dependency with respect to their peers is only half the size both for childless women and for mothers than in all the other countries (where it is around or over a third of a standard deviation). A possible explanation for that may be existence of a more modern manufacture process and less gendered norms in those sectors in those countries than in the rest of Europe (Matysiak et al., 2023). Conversely, in Eastern Europe the gender gap in the use of machinery among parents is the largest.

**Table 3:** Female coefficient in prevalence models with full controls by country groups and parenthood status

	(1) Contact	(2) Autonomy	(3) Time pressure	(4) Leadership	(5) Machine-dependent
*Continental*					
All [11,460]	0.22*** (0.026)	− 0.10*** (0.025)	− 0.15*** (0.025)	− 0.31*** (0.023)	− 0.39*** (0.021)
Parents [7627]	0.16*** (0.032)	− 0.19*** (0.031)	− 0.18*** (0.029)	− 0.40*** (0.032)	− 0.39*** (0.027)
Childless [3833]	0.33*** (0.034)	0.05 (0.042)	− 0.10** (0.041)	− 0.15*** (0.035)	− 0.39*** (0.032)
*Eastern*					
All [13,239]	0.11*** (0.026)	− 0.14*** (0.028)	− 0.13*** (0.031)	− 0.33*** (0.033)	− 0.38*** (0.025)
Parents [8253]	0.07** (0.034)	− 0.16*** (0.038)	− 0.12*** (0.039)	− 0.39*** (0.039)	− 0.41*** (0.033)
Childless [4986]	0.18*** (0.042)	− 0.05 (0.038)	− 0.10** (0.049)	− 0.19*** (0.045)	− 0.34*** (0.042)
*Southern*					
All [5146]	0.06* (0.031)	− 0.17*** (0.032)	− 0.07** (0.033)	− 0.35*** (0.034)	− 0.34*** (0.027)
Parents [3028]	0.01 (0.039)	− 0.23*** (0.050)	− 0.05 (0.045)	− 0.35*** (0.048)	− 0.34*** (0.035)
Childless [2118]	0.16*** (0.051)	− 0.09** (0.048)	− 0.08 (0.054)	− 0.34*** (0.044)	− 0.37*** (0.042)
*UK*					
All [2722]	0.19*** (0.043)	0.01 (0.041)	− 0.16** (0.045)	− 0.13*** (0.051)	− 0.22*** (0.043)
Parents [1716]	0.18*** (0.054)	0.00 (0.059)	− 0.18*** (0.063)	− 0.17*** (0.060)	− 0.25*** (0.48)
Childless [1006]	0.19*** (0.060)	0.02 (0.052)	− 0.11* (0.059)	− 0.04 (0.069)	− 0.14** (0.061)
*Denmark*					
All [3897]	0.13*** (0.033)	− 0.03 (0.034)	− 0.02 (0.035)	− 0.19*** (0.038)	− 0.26*** (0.031)
Parents [3072]	0.14*** (0.037)	− 0.03 (0.042)	− 0.03 (0.037)	− 0.19*** (0.042)	− 0.26*** (0.040)
Childless [825]	0.06 (0.069)	− 0.06 (0.067)	− 0.01 (0.087)	− 0.19** (0.083)	− 0.21*** (0.066)

Weighted analytic sample. All models include controls for age, age squared, education, public, temporary, sector at the ISIC 2-digit level, and country fixed effects within groups. See text for country groups. Parents defined as responding yes to: “Do you have a child?”

Sample sizes in square brackets []

Paired jackknife standard errors in parentheses ***p < 0.01; **p < 0.05; *p < 0.1

Leadership is much more gendered among parents everywhere compared to UK and Denmark and the gaps in leadership are even as large also for non-mothers in Southern Europe compared to childless men. Similarly, mothers have much less autonomy in their positions than fathers in Continental, Eastern and Southern Europe and again the gender difference in autonomy among the childless is only sizable in Southern Europe. Interestingly in Southern Europe, gender differences in time pressure among parents are not significant.

## Wage Returns to Occupational Characteristics by Gender and Parenthood

In Table 4, we use log hourly wages to estimate, first, the return to occupational characteristics; and second, whether or not women receive a differential return by interacting the characteristics with the female indicator. Table 4 presents estimates for all workers and separately for childless and parents (with children of different ages) controlling for basic demographic characteristics, country and sector of employment.

**Table 4:** Full wage models with and without female interactions by parenthood and age of the child

	All workers		Childless only		Parents only		Parents with a child less than 12 years old		Parents with a child less than 18 years old	
	(1)	(2)	(3)	(4)	(5)	(6)	(9)	(10)	(11)	(12)
Female	− 0.14***	− 0.16***	− 0.18**	− 0.18*	− 0.11**	− 0.12**	− 0.15***	− 0.17***	− 0.13***	− 0.16***
	(0.046)	(0.052)	(0.088)	(0.096)	(0.050)	(0.055)	(0.044)	(0.035)	(0.035)	(0.031)
Secondary education	0.13**	0.13**	0.12	0.12	0.14**	0.14**	0.17***	0.17***	0.18***	0.17***
	(0.059)	(0.057)	(0.096)	(0.097)	(0.056)	(0.055)	(0.050)	(0.049)	(0.038)	(0.038)
Tertiary education	0.37***	0.37***	0.24**	0.24*	0.45***	0.46***	0.43***	0.43***	0.45***	0.46***
	(0.081)	(0.082)	(0.115)	(0.119)	(0.076)	(0.078)	(0.059)	(0.061)	(0.052)	(0.055)
Public sector	0.04	0.04	0.14	0.13	0.03	0.03	0.02	0.03	− 0.05	− 0.04
	(0.054)	(0.054)	(0.103)	(0.102)	(0.063)	(0.062)	(0.038)	(0.037)	(0.057)	(0.056)
Temporary contract	− 0.16***	− 0.16***	− 0.18**	− 0.17**	− 0.15**	− 0.15**	− 0.00	− 0.00	− 0.06	− 0.06
	(0.041)	(0.041)	(0.071)	(0.071)	(0.058)	(0.059)	(0.083)	(0.084)	(0.066)	(0.067)
Contact	− 0.10**	− 0.12**	− 0.14*	− 0.15	− 0.07	− 0.10**	− 0.07**	− 0.10**	− 0.09**	− 0.13***
	(0.043)	(0.051)	(0.084)	(0.126)	(0.048)	(0.041)	(0.032)	(0.041)	(0.036)	(0.033)
Female#contact		0.03		0.01		0.04		0.07*		0.08
		(0.091)		(0.155)		(0.089)		(0.039)		(0.063)
Autonomy	0.08**	0.10**	0.10*	0.13*	0.06*	0.08*	0.04	0.04	0.05**	0.07
	(0.034)	(0.041)	(0.057)	(0.079)	(0.035)	(0.043)	(0.031)	(0.053)	(0.022)	(0.044)
Female#autonomy		− 0.05		− 0.08		− 0.05		− 0.01		− 0.04
		(0.057)		(0.096)		(0.074)		(0.062)		(0.071)
Time pressure	0.01	− 0.03	− 0.02	− 0.08	0.02	0.02	0.03	0.02	0.02	0.02
	(0.019)	(0.039)	(0.039)	(0.068)	(0.022)	(0.047)	(0.034)	(0.077)	(0.026)	(0.060)
Female#time pressure		0.06		0.13		− 0.00		0.01		0.00
		(0.047)		(0.087)		(0.056)		(0.077)		(0.064)
Leadership	0.09***	0.13***	0.08*	0.10	0.10***	0.14***	0.12***	0.18***	0.10***	0.16***
	(0.022)	(0.039)	(0.042)	(0.068)	(0.029)	(0.044)	(0.029)	(0.057)	(0.029)	(0.044)
Female#leadership		− 0.06		− 0.01		− 0.08		− 0.12*		− 0.10*
		(0.048)		(0.076)		(0.055)		(0.068)		(0.061)
Machine-dependent	− 0.10***	− 0.09***	− 0.18***	− 0.18**	− 0.06**	− 0.05	− 0.07*	− 0.05	− 0.10***	− 0.07*
	(0.030)	(0.035)	(0.065)	(0.083)	(0.030)	(0.032)	(0.035)	(0.051)	(0.032)	(0.034)
Female#machine		− 0.02		0.01		− 0.04		− 0.04		− 0.11**
		(0.048)		(0.105)		(0.049)		(0.052)		(0.051)
Constant	1.70***	1.71***	1.41***	1.44***	1.60***	1.59***	2.24***	2.21***	1.58***	1.56***
	(0.319)	(0.318)	(0.437)	(0.437)	(0.475)	(0.473)	(0.599)	(0.600)	(0.477)	(0.484)
Observations	36,464	36,464	12,768	12,768	23,696	23,696	11,392	11,392	15,377	15,377
R-squared	0.082	0.082	0.081	0.081	0.094	0.094	0.137	0.138	0.110	0.112

Weighted analytic sample. All models include controls for age, age squared, sector at the ISIC 2-digit level, and country fixed effects. Parents defined as responding yes to: “Do you have a child?.” Last four columns include parents who have at least one child under the indicated age

Paired jackknife standard errors in parentheses ***p < 0.01; **p < 0.05; *p < 0.1

Overall women make around 0.14–0.16 log points less in hourly wages than comparable men in columns (1–2). The basic gap is not larger for mothers in columns (5–6) compared to childless women in columns (3–4) suggesting that mothers working more than 20 h are potentially positively selected. This may also explain that the positive return for education is the highest among parents and the lowest among childless individuals. Wages are not significantly different in public sector jobs compared to private sector ones, which may favour women who are overrepresented in the former. The wage penalty in temporary contracts is high except for parents of young children. A possible explanation is that only those with sufficiently high wages despite holding a temporary contract become parents and/or stay in the labour force.

With regard to occupational requirements, wages in contact-intense jobs tend to be lower. While the interaction of female and contact is positive, it is only marginally significant (at 10%) for mothers of children under 12. As expected, autonomy is positively rewarded and there are no gender differences in the return. There is no premium for jobs requiring time pressure, not even for women who, we posited in the theory section, might expect a higher return for choosing those positions.

The most distinctive gender differences emerge in the returns to the last two factors: leadership and machine dependency. Leadership is highly rewarded especially for fathers, while mothers in leadership positions are penalized, but the interaction coefficient is only marginally significant for those with minors. The largest gender gap in the returns to leadership appears among parents of children under 12, for whom the return to fathers for each standard deviation of leadership requirements is 0.18 log point, but that gain shrinks by 0.13 log points for mothers. It is important to keep in mind, that mothers’ occupations display the lowest prevalence in leadership to start with, so even those who are selected into those positions do not receive equal compensation for their performance.

Finally, occupations with high machine dependency are penalized for all workers and even more for childless individuals. Seniority rules and stratification within factory floors might be more important in machine-intensive sectors than in others. In addition, there is a double penalty for mothers in machine-oriented jobs (only significant for mothers with children under 18).

In robustness analyses, we use log hourly wages including bonuses and find similar results (Appendix Table 12).

### Wage Returns to Occupational Characteristics by Gender and Parenthood: Country Differences

Table 5 presents the models with gender interactions across country groups for all workers and for parents and Appendix Table 13 for childless and parents with young children. With all controls included, the remaining gender wage penalty is large and significant everywhere except for Continental Europe.

**Table 5:** Wage models with female interaction by parenthood and country groups

	Continental		Eastern		Southern		UK		Denmark	
	(1)	(2)	(3)	(4)	(5)	(6)	(7)	(8)	(9)	(10)
	All workers	Parents	All workers	Parents	All workers	Parents	All workers	Parents	All workers	Parents
Female	− 0.15	− 0.05	− 0.17***	− 0.19***	− 0.12***	− 0.17***	− 0.15***	− 0.19***	− 0.08***	− 0.08***
	(0.124)	(0.118)	(0.016)	(0.018)	(0.016)	(0.018)	(0.020)	(0.028)	(0.015)	(0.019)
Secondary education	0.18	0.17	0.10**	0.07**	0.10***	0.08***	0.13***	0.10***	0.19***	0.13**
	(0.115)	(0.119)	(0.047)	(0.035)	(0.020)	(0.023)	(0.036)	(0.036)	(0.036)	(0.050)
Tertiary education	0.38**	0.52***	0.36***	0.37***	0.31***	0.35***	0.33***	0.32***	0.37***	0.32***
	(0.166)	(0.159)	(0.052)	(0.047)	(0.023)	(0.029)	(0.047)	(0.049)	(0.039)	(0.055)
Public sector	0.02	− 0.01	0.04	0.05	0.12***	0.14***	0.11***	0.13***	− 0.03	− 0.01
	(0.104)	(0.112)	(0.026)	(0.033)	(0.024)	(0.029)	(0.035)	(0.036)	(0.021)	(0.026)
Temporary contract	− 0.21*	− 0.17	− 0.11***	− 0.11***	− 0.12***	− 0.12***	− 0.08***	− 0.12***	− 0.10***	− 0.05*
	(0.097)	(0.140)	(0.018)	(0.023)	(0.018)	(0.024)	(0.028)	(0.034)	(0.025)	(0.028)
Contact	− 0.20*	− 0.15	− 0.09***	− 0.10***	0.00	− 0.02	− 0.09***	− 0.12***	− 0.05*	− 0.06*
	(0.116)	(0.094)	(0.017)	(0.019)	(0.018)	(0.021)	(0.028)	(0.037)	(0.024)	(0.031)
Female#contact	0.02	0.09	0.02	0.01	− 0.02	− 0.01	0.07*	0.09**	0.02	0.03
	(0.210)	(0.192)	(0.026)	(0.031)	(0.023)	(0.027)	(0.039)	(0.044)	(0.026)	(0.032)
Autonomy	0.16*	0.11	0.06***	0.07***	0.03*	0.04*	0.08**	0.12***	0.07***	0.07***
	(0.084)	(0.080)	(0.016)	(0.021)	(0.016)	(0.023)	(0.033)	(0.039)	(0.019)	(0.026)
Female#autonomy	− 0.09	− 0.12	− 0.00	− 0.01	− 0.02	− 0.02	− 0.08*	− 0.10**	− 0.04*	− 0.05
	(0.122)	(0.151)	(0.024)	(0.032)	(0.023)	(0.031)	(0.040)	(0.046)	(0.021)	(0.029)
Time pressure	− 0.06	0.05	0.00	0.00	− 0.01	0.02	0.03	− 0.00	− 0.02	− 0.01
	(0.086)	(0.101)	(0.012)	(0.013)	(0.011)	(0.013)	(0.021)	(0.027)	(0.019)	(0.027)
Female#time pressure	0.11	− 0.02	0.02*	0.03**	0.01	− 0.02	0.03	0.08**	0.02	0.01
	(0.102)	(0.122)	(0.013)	(0.015)	(0.015)	(0.016)	(0.025)	(0.030)	(0.021)	(0.029)
Leadership	0.19**	0.20**	0.08***	0.08***	0.06***	0.06***	0.10***	0.10***	0.05***	0.04***
	(0.074)	(0.087)	(0.014)	(0.017)	(0.014)	(0.018)	(0.024)	(0.025)	(0.012)	(0.014)
Female#leadership	− 0.13	− 0.16	0.03*	0.03	0.02	− 0.00	0.01	0.02	− 0.01	0.01
	(0.095)	(0.107)	(0.017)	(0.021)	(0.015)	(0.021)	(0.031)	(0.037)	(0.014)	(0.015)
Machine-dependent	− 0.11	− 0.00	− 0.12***	− 0.11***	− 0.06***	− 0.08***	− 0.13***	− 0.12***	− 0.06***	− 0.07***
	(0.075)	(0.062)	(0.015)	(0.017)	(0.013)	(0.017)	(0.019)	(0.023)	(0.015)	(0.019)
Female#machine	− 0.03	− 0.05	− 0.04*	− 0.05**	− 0.02	− 0.01	0.02	− 0.02	− 0.02	− 0.02
	(0.098)	(0.100)	(0.019)	(0.020)	(0.021)	(0.025)	(0.029)	(0.035)	(0.019)	(0.023)
Constant	1.88***	1.35	1.51***	1.71***	1.07**	1.07***	1.14***	1.45***	1.34***	1.71***
	(0.568)	(0.824)	(0.150)	(0.237)	(0.128)	(0.203)	(0.109)	(0.193)	(0.195)	(0.344)
Observations	11,462	7627	13,246	8253	5146	3028	2723	1716	3899	3072
R-squared	0.043	0.051	0.309	0.354	0.394	0.487	0.515	0.494	0.332	0.237

Weighted analytic sample. All models include controls for age, age squared, education, public, temporary, sector at the ISIC 2-digit level, and country fixed effects within groups. Parents defined as responding yes to: “Do you have a child?.” See text for country groups

Paired jackknife standard errors in parentheses ***p < 0.01; **p < 0.05; *p < 0.1

Jobs with high contact requirements are penalized everywhere except in Southern Europe. For all women and mothers in the UK the penalty from contact intensive occupations is significantly lower than for men. Conversely, autonomy is rewarded everywhere but among UK women and mothers.

While in Continental Europe leadership is rewarded the most across all groups, the interaction of female and leadership is negative and large (especially in the parental sample), but not significant. It is highly significant, though, when restricting the sample to parents with children either under 18 or under 12 (Table 13) indicating that mothers of young children in Continental Europe are in fact penalized when holding leadership positions compared to fathers. Conversely, Eastern European women (but not mothers) are marginally rewarded for leadership positions. The return to time pressure is only marginally significant among women in Eastern Europe and mothers in UK and Denmark. Jobs requiring the use of machines are penalized across except in Continental Europe. Eastern European women in machine-intense occupations receive a double penalty.

## Oaxaca–Blinder Decomposition of Gender Wage Gaps

Table 6 presents estimates of an Oaxaca–Blinder decomposition of the gender wage gap to study whether the contrasting prevalence of the five occupational factors across gender and parenthood shown in Tables 2 and 3 can account for part of the observed overall differences in log hourly wages. The first four columns include models for all workers and for childless respondents, with and without controlling for O*NET factors. The remaining columns focus on parents and the five occupational characteristics are included one by one in Columns (7) to (11) to see how each contributes separately.

**Table 6:** Oaxaca–Blinder decomposition by gender and parenthood: contribution of individual O*NET factors

	All		Childless		Parents						
	(1)	(2)	(3)	(4)	(5)	(6)	(7)	(8)	(9)	(10)	(11)
	No O*NET	All controls	No O*NET	All controls	No O*NET	All controls	Contact	Autonomy	Time pressure	Leadership	Machine-dependent
Men average	2.77*** (0.030)		2.63*** (0.051)		2.86*** (0.037)						
Women average	2.63*** (0.038)		2.60*** (0.061)		2.64*** (0.038)						
Gender differences	0.15*** (0.038)		0.03 (0.066)		0.22*** (0.048)						
Explained	− 0.01	0.00	− 0.14***	− 0.15***	0.08**	0.11**	0.07**	0.09***	0.09***	0.11***	0.06*
	(0.022)	(0.030)	(0.039)	(0.046)	(0.030)	(0.046)	(0.031)	(0.030)	(0.030)	(0.035)	(0.037)
Unexplained	0.16***	0.14***	0.17*	0.18**	0.14***	0.11**	0.15***	0.13**	0.13***	0.11**	0.16***
	(0.046)	(0.046)	(0.088)	(0.088)	(0.050)	(0.050)	(0.049)	(0.049)	(0.049)	(0.049)	(0.052)
*Explained*											
Age	− 0.00	− 0.00	0.00	− 0.00	0.00	0.00	0.00	0.00	0.00	0.00	0.00
	(0.003)	(0.002)	(0.004)	(0.003)	(0.004)	(0.002)	(0.004)	(0.004)	(0.004)	(0.004)	(0.004)
Education	− 0.03***	− 0.02***	− 0.04**	− 0.02*	− 0.02***	− 0.02***	− 0.02***	− 0.02***	− 0.02***	− 0.02***	− 0.02***
	(0.005)	(0.005)	(0.011)	(0.012)	(0.004)	(0.004)	(0.004)	(0.004)	(0.004)	(0.004)	(0.004)
Public sector	− 0.01	− 0.01	− 0.02	− 0.02	− 0.01	− 0.00	− 0.01	− 0.00	− 0.01	− 0.00	− 0.01
	(0.008)	(0.008)	(0.012)	(0.012)	(0.010)	(0.010)	(0.010)	(0.010)	(0.010)	(0.010)	(0.010)
Temporary contract	0.00*	0.00*	0.00	0.00	0.00**	0.00**	0.00**	0.00**	0.00**	0.00**	0.00**
	(0.001)	(0.001)	(0.002)	(0.002)	(0.001)	(0.001)	(0.001)	(0.001)	(0.001)	(0.001)	(0.001)
Sector	0.01	0.02	− 0.07*	− 0.05	0.07**	0.08***	0.08***	0.08**	0.06**	0.08***	0.08***
	(0.020)	(0.020)	(0.040)	(0.039)	(0.029)	(0.028)	(0.028)	(0.029)	(0.030)	(0.030)	(0.027)
Country	0.01**	0.01**	− 0.01	− 0.01	0.02***	0.02***	0.02***	0.02***	0.02***	0.02***	0.02***
	(0.005)	(0.005)	(0.012)	(0.012)	(0.007)	(0.007)	(0.007)	(0.007)	(0.007)	(0.007)	(0.007)
Contact		0.04**		0.08*		0.02	− 0.01*				
		(0.017)		(0.045)		(0.015)	(0.008)				
Autonomy		0.00		− 0.02		0.01		0.01***			
		(0.001)		(0.009)		(0.004)		(0.003)			
Time pressure		0.00		− 0.00		0.01			0.02***		
		(0.006)		(0.011)		(0.008)			(0.007)		
Leadership		0.01***		0.00		0.02***				0.02***	
		(0.004)		(0.003)		(0.007)				(0.006)	
Machine		− 0.06***		− 0.11***		− 0.03**					− 0.02
		(0.017)		(0.041)		(0.016)					(0.016)
Observations	36,464	36,464	12,768	12,768	23,696	23,696	23,696	23,696	23,696	23,696	23,696

Weighted analytic sample. Only coefficients for explained part reported. Parents defined as responding yes to: “Do you have a child?”

Paired jackknife standard errors in parentheses ***p < 0.01; **p < 0.05; *p < 0.1

While the observed average wage gap is substantial and significant among parents, in the order of 0.22 log points, it amounts to an insignificant 0.03 log points among childless workers. Columns (1), (3), and (5) present models with the basic controls. As expected, educational variables always enter negatively in the explained part meaning that higher educational achievement favours women: it decreases the observed wage gap for mothers (column 5) or can even lead to equality of pay on its own in the childless sample (column 3). While public sector is not significant, the high prevalence and low return of temporary work hurts mothers but only accounts for a minimal part of the gap. Country differences are important for parents (close to 10% of the gap), but not for childless individuals and they may relate to differences in the institutional settings or gender norms across these countries with regard to motherhood employment that cannot be fully captured with our variables. Finally, sectoral differences explain a large share of the wage gap among parents (close to one third of it) in column (5) meaning mothers sort into sectors with lower wages than those of fathers. Sectoral composition works in the opposite direction for childless women (in column 3), meaning that their wages should be higher compared to those of childless men considering the sectors in which they work.

When the five occupational characteristics are included in column (6), the explained part for mothers moves up from 0.08 log points to 0.11 out of the 0.22 log points difference, implying the variables in the model explain around 50% of the gap. Conversely, more is left unexplained now for why wages of childless women are not closer to men’s in column (4). Recent related work in Europe has also shown that large parts of the variation cannot be explained (Christofides et al., 2013; Redmond & McGuinness, 2019).

Differences in leadership and the high reward to these skills appear as a significant factor to explain the wage gap among parents (around 10%). Gender differences in the use of machines at work are relevant for everyone, especially the childless, and should imply lower wage gaps than observed. Higher contact and the relatively lower reward to this occupational characteristic increase the gap among childless.

Results are slightly different when adding occupational factors one by one instead of pooling them together in the model. The larger differences appear, first, in column (7) when contact changes its sign to marginally imply their prevalence should favour mothers and second, in columns (8) and (9) where both autonomy and time pressure are now highly significant and explain a relevant part of the motherhood gap on their own (5 to 10%). Their lack of significance in the model with all controls in column (6) is likely related to the fact that, as discussed in the theoretical section, autonomy is moderately correlated with leadership and contact (see Table 9 in Appendix).

### Oaxaca–Blinder Decomposition of Gender Wage Gaps: Country Differences

Table 7 presents similar Oaxaca–Blinder decomposition models separately for each country group.^6^ The gap is always larger among parents than for the whole sample. Except for Continental countries, the net share of the gap explained by the occupational factors is very small. Even though most of them are significant, they work in opposite directions with machine use decreasing the part explained. Sectoral differences are very important and explain between 33 and 50% of the gap among parents in all country groups.

**Table 7:** Full Oaxaca–Blinder decomposition by gender, parenthood, and country groups

	Continental		Eastern		Southern		UK		Denmark	
	(1)	(2)	(3)	(4)	(5)	(6)	(7)	(8)	(9)	(10)
	All	Parent	All	Parent	All	Parent	All	Parent	All	Parent
Men average	3.08***	3.17***	2.09***	2.13***	2.55***	2.63***	2.84***	2.92***	3.14***	3.23***
	(0.063)	(0.075)	(0.013)	(0.016)	(0.011)	(0.014)	(0.015)	(0.019)	(0.011)	(0.013)
Women average	2.91***	2.92***	1.94***	1.94***	2.44***	2.47***	2.70***	2.73***	3.07***	3.11***
	(0.078)	(0.075)	(0.013)	(0.016)	(0.013)	(0.014)	(0.019)	(0.023)	(0.011)	(0.010)
Gender difference	0.17**	0.25**	0.15***	0.19***	0.11***	0.16***	0.14***	0.19***	0.07***	0.12***
	(0.077)	(0.095)	(0.018)	(0.022)	(0.017)	(0.021)	(0.025)	(0.026)	(0.016)	(0.017)
Explained	0.04	0.21**	− 0.02	0.01	− 0.02	0.00	− 0.00	0.02	− 0.01	0.04***
	(0.069)	(0.100)	(0.014)	(0.019)	(0.012)	(0.016)	(0.023)	(0.026)	(0.012)	(0.013)
Unexplained	0.13	0.04	0.17***	0.18***	0.12***	0.16***	0.14***	0.17***	0.07***	0.08***
	(0.099)	(0.101)	(0.016)	(0.018)	(0.015)	(0.018)	(0.018)	(0.030)	(0.014)	(0.017)
*Explained*										
Contact	0.08*	0.03	0.04***	0.03***	0.00	0.00	0.02***	0.03**	0.01**	0.01**
	(0.042)	(0.033)	(0.007)	(0.007)	(0.004)	(0.003)	(0.006)	(0.010)	(0.006)	(0.007)
Autonomy	0.00	0.01	− 0.00	0.01**	0.00	0.01*	− 0.00	− 0.00	− 0.00*	− 0.00
	(0.003)	(0.009)	(0.002)	(0.003)	(0.001)	(0.004)	(0.002)	(0.003)	(0.002)	(0.002)
Time pressure	0.00	0.02	0.00	0.01*	0.00	0.00	0.02***	0.02***	− 0.00	0.00
	(0.014)	(0.017)	(0.002)	(0.003)	(0.002)	(0.003)	(0.005)	(0.006)	(0.003)	(0.004)
Leadership	0.02***	0.03**	0.01***	0.02***	0.01***	0.01***	− 0.00	− 0.00	− 0.01***	− 0.01***
	(0.008)	(0.016)	(0.004)	(0.004)	(0.003)	(0.003)	(0.005)	(0.005)	(0.002)	(0.002)
Machine	− 0.07*	− 0.01	− 0.09***	− 0.08***	− 0.04***	− 0.05***	− 0.05***	− 0.06***	− 0.03***	− 0.03***
	(0.035)	(0.029)	(0.009)	(0.010)	(0.007)	(0.008)	(0.008)	(0.011)	(0.006)	(0.007)
Age	− 0.00	0.00	− 0.01***	− 0.00	− 0.00	0.01**	− 0.01	− 0.00	− 0.02***	− 0.00
	(0.003)	(0.004)	(0.001)	(0.001)	(0.003)	(0.002)	(0.007)	(0.003)	(0.005)	(0.003)
Education	− 0.01*	− 0.00	− 0.03***	− 0.02***	− 0.03***	− 0.03***	− 0.01*	− 0.01	− 0.01***	− 0.00
	(0.005)	(0.003)	(0.004)	(0.006)	(0.005)	(0.006)	(0.006)	(0.006)	(0.004)	(0.004)
Public	− 0.00	0.00	− 0.01*	− 0.01	− 0.01***	− 0.01***	− 0.02***	− 0.04***	0.01	0.00
	(0.013)	(0.015)	(0.006)	(0.007)	(0.003)	(0.004)	(0.008)	(0.012)	(0.007)	(0.008)
Temporary	0.00	0.00	− 0.00	0.00	0.00**	0.01***	− 0.00	− 0.00*	0.00	0.00
	(0.002)	(0.003)	(0.002)	(0.002)	(0.002)	(0.002)	(0.001)	(0.003)	(0.001)	(0.001)
Sector	− 0.00	0.09*	0.06***	0.06***	0.05***	0.06***	0.04***	0.08***	0.05***	0.06***
	(0.041)	(0.054)	(0.011)	(0.014)	(0.009)	(0.013)	(0.015)	(0.023)	(0.011)	(0.013)
Country	0.02***	0.02***	0.00***	0.00***	0.00*	0.00				
	(0.006)	(0.009)	(0.001)	(0.001)	(0.002)	(0.004)				
Observations	11,460	7627	13,239	8253	5146	3028	2722	1716	3897	3072

Weighted analytic sample. Only coefficients for explained part reported. Parents defined as responding yes to: “Do you have a child?.” See text for country groups

Paired jackknife standard errors in parentheses ***p < 0.01; **p < 0.05; *p < 0.1

In continental countries, the combination of all variables is sizable for parents: out of 0.25 log points of difference, 0.21 are explained (0.09 alone from sectoral differences). Mothers are heavily penalized for the lower prevalence of leadership positions and its high associated return. In Eastern Europe, four occupational characteristics (contact, autonomy, time pressure and leadership) significantly contribute to explaining the gap among parents, but machine use and education balance out in the opposite direction. In Southern Europe, leadership is the most relevant factor jointly with sectoral differences to explain the gap, while heavy machine use by fathers works in the opposite direction.

In the UK, out of 0.19 log points of gap among parents, around 0.02 log point are accounted for our variables and in Denmark, 0.4 log points are significantly explained out of 0.12 points of gender gap. More contact and less autonomy hurt mothers in the UK, while leadership works in the opposite direction as elsewhere in Denmark. In both countries the sector of employment is highly relevant (close to 50%).

## Discussion and Conclusion

Despite important advances in the closing of gender wage gaps in European countries, gender differences in the type of work men and women undertake and the reward they receive continues to be pervasive, particularly among parents. In this paper, we isolate a set of occupational characteristics (contact with others, autonomy, time pressure, leadership and machine use) whose prevalence and wage returns, we argue, are likely to differ by gender and may account for some of the remaining differences. The paper sets to answer three questions on the gendered nature of work.

First, the relative prevalence by gender and parenthood status of the five occupational characteristics studied generally aligns with our expectations. Among them, only contact with others is, on average, more prevalent in jobs held by women and mothers in particular, while machine use and leadership are much more prevalent in men’s jobs. But, whereas there is no difference in machine use between parents and childless men, fathers emerge as the ones holding leadership roles, in line with previous works on the existence of both a glass ceiling for women and a fatherhood premium (Arulampalam et al., 2007; Christofides et al., 2013). Differences in time pressure are not substantial, but the gap in autonomy is large among parents. Even though we did not have a clear expectation in terms of the overall gender gap for the latter, the sizable autonomy of fathers’ jobs might be consistent with them being more associated with managerial positions than those of younger (childless) workers.

While these results hold in all countries, the gender gap in leadership is particularly large in Southern Europe and between mothers and fathers in Eastern and Continental Europe. Southern and Eastern Europe also display gender gaps in autonomy, while Continental Europe, UK, and Denmark do not. Taken together, the prevalence findings are indicative of more gender-based occupational sorting in Eastern and Southern Europe.

Second, we find persistent gender wage gaps even after controlling for sector, demographic, and occupational characteristics, with the possible exception of Continental Europe. Overall, hourly wages for women (and marginally more for mothers of young children) are lower than those of similar men. This is partially explained by the fact that occupations with more contact, where women are more prevalent, are associated with lower wages everywhere except in Southern Europe. This finding does not align with the expectation that jobs may be rewarded when contact involves a personalized relationship as in Goldin (2014), but rather with a more gendered story of contact jobs being more prevalent among women, in line with Levanon and Grusky (2016). While autonomy is positively and equally rewarded for all, even though fathers are relatively more represented in autonomy-intensive jobs; (male) leadership is highly rewarded, especially among fathers compared to mothers with young children. Partially compensating the male advantage, machine-dependent occupations have low returns. Strikingly, there is an additional wage penalty for women working in these occupations in Eastern Europe.

The third question we ask is whether the different prevalence of those characteristics combined with their return can account for the observed gender gap in hourly wages. Oaxaca–Blinder decomposition models show that both the current educational advantage of women and men holding more machine-intensive occupations partially close the gap. At the same time, less leadership positions for women account for a sizable part of the gap, around 10% among parents. In line with the findings in Blau and Kahn (2017) of an increasing relevance in sectoral sorting in the US data, sectoral differences account for 33–50% of the wage gap among parents in all country groups. Overall, the (net) share explained in Oaxaca–Blinder models including all women (mothers and non-mothers) is not meaningful because of the opposing above-mentioned forces. This is consistent with recent research in Europe showing that a large part of the gender gap in Europe remains unexplained (Christofides et al., 2013; Redmond & McGuinness, 2019). Even though these same variables explain a sizable share of the gap among parents, around 50% remains unexplained. Across country groups, they explain the most in Continental Europe and Denmark and the least in Eastern Europe. Further, even though our focus is on parents, we highlight that, while the gap for childless individuals is generally small, Oaxaca–Blinder decomposition models show that, considering current endowments and sectoral and occupational choices, those gaps should be larger and favour women.

These findings provide insights about future trends in the gender wage gap in light of long-term changes in the labour market, such as outsourcing and technological changes (Cortes et al., 2020). If European economies continue shifting from manufacturing to service jobs that cannot be outsourced, in the long run women might benefit more than men from increased demand in occupations that tend to require higher contact with others. However, the extent to which many of these service occupations that require high social skills, an attribute associated with women (Cejka & Eagly, 1999; Levanon & Grusky, 2016), continue to be poorly paid (i.e. care and social services) may limit the closing of the gender gap (England, 2010). A boost in policies promoting women in leadership could further enhance the closing, but this is unlikely to substantially increase women’s standing unless it is coupled with increased autonomy in control over both tasks and schedules. These changes are likely to occur differentially for women working in low- vs. high-skilled occupations (Matysiak et al., 2023). For men in countries with widespread adoption of advanced technologies, the changing nature of machine-dependent occupations will likely shift the balance transforming low-skilled occupations that are currently low paid to highly specialized and well-paid machine operators (in some cases requiring STEM credentials), thus pushing for a widening of the gender wage gap, at least, among high-skilled workers.

Our findings and some of the limitations of the data should inform future analysis. First, as we can only observe the final equilibrium in the labour market, the distribution of occupational characteristics observed in the data has already been negotiated by workers given differences in the availability of jobs with different characteristics. If computerization, for example, sharply decreases the demand for manual jobs requiring the use of machinery, we should expect to observe fewer of those jobs at the aggregate level. The degree to which those changes have occurred across the different countries in our sample may contribute to the differential gender prevalence of occupational characteristics such as machine-intense use. We found those gaps to be substantially smaller in Denmark or the UK and noted that a more rapid adaptation of manufacturing to new IT opportunities in those countries may be a mechanism to explain that pattern.

Second, an important limitation is that the analysis of occupational differences is limited to the working population and there may be differential drop out of the labour market across Europe, especially at motherhood. German women are among those more likely to drop around childbirth and take longer to return (Kleven et al., 2019a, 2019b). In Southern Europe the difficulties of obtaining part-time arrangements (compared to places like Continental Europe or Nordic countries) may determine the extent of selection among mothers remaining in the market. Family policies and generous leaves that allow mothers to remain attached to the market and minimize human capital depreciation contribute to a large extent to these cross-country differences (Olivetti & Petrongolo, 2017; Thévenon, 2011). At the same time some of these policies may result in more sorting of women in service and care occupations, many of them in the public sector as they constitute more secure positions. In countries with lower inequality like Denmark this may result in more moderate gaps than in others in which some of these jobs are more penalized.

Third, our measures cannot capture firm-specific policies that may affect the meaning of our occupational characteristics. For example, larger firms may be able to provide family reconciliation policies than small firms, for which those policies may be too costly. Those policies may ease up, at least temporarily, time pressure and contact requirements. In Southern Europe, the relatively small size of firms implies a very low coverage of these policies. Only around 20% of firms in Greece and around one third of those in Spain provides them in 2019 compared to 77% in Denmark or 55% in France (Eurostat 2019). Commuting cost which has been noted as an important job characteristic to explain gender gaps recently is something our data cannot capture as is clearly firm- and location-specific (Petrongolo & Ronchi, 2020). Finally, an important extension of this analysis would be to consider intra-family dynamics in the choices of occupations and how the extent of specialization has been evolving over time with the transformations of the labour market. For that undertaking, either a rich panel dataset with household and occupational information or family longitudinal data would be needed.

## Appendix

See Tables 8, 9, 10, 11, 12 and 13.

**Table 8:** Items included in the variables capturing contact with others, autonomy, leadership, machine dependency, and time pressure

Element ID	Variable name	Description	Factor loading
(A) *Contact with others*			
4A4a2	Communicating with supervisors, peers, or subordinates	Providing information to supervisors, co-workers, and subordinates by telephone, in written form, e-mail, or in person	0.65
4A4a3	Communicating with people outside the organization	Communicating with people outside the organization, representing the organization to customers, the public, government, and other external sources. This information can be exchanged in person, in writing, or by telephone or e-mail	0.86
4A4a4	Establishing and maintaining interpersonal relationships	Developing constructive and cooperative working relationships with others, and maintaining them over time	0.85
4A4a8	Performing for or working directly with the public	Performing for people or dealing directly with the public. This includes serving customers in restaurants and stores, and receiving clients or guests	0.64
4C1a4	Contact with others	How much does this job require the worker to be in contact with others (face-to-face, by telephone, or otherwise) in order to perform it?	0.83
4C1b1e	Work with work group or team	How important is it to work with others in a group or team in this job?	0.60
4C1b1f	Deal with external customers	How important is it to work with external customers or the public in this job?	0.84
(B) *Leadership*			
4A4b1	Coordinating the work and activities of others	Getting members of a group to work together to accomplish tasks	0.93
4A4b2	Developing and building teams	Encouraging and building mutual trust, respect, and cooperation among team members	0.92
4A4b3	Training and teaching others	Identifying the educational needs of others, developing formal educational or training programmes or classes, and teaching or instructing others	0.80
4A4b4	Guiding, directing, and motivating subordinates	Providing guidance and direction to subordinates, including setting performance standards and monitoring performance	0.93
4A4b5	Coaching and developing others	Identifying the developmental needs of others and coaching, mentoring, or otherwise helping others to improve their knowledge and skills	0.94
4A4b6	Providing consultation and advice to others	Providing guidance and expert advice to management or other groups on technical systems-, or process-related topics	0.77
(C) *Autonomy in decisions*			
4C3a4	Freedom to make decisions	How much decision-making freedom, without supervision, does the job offer?	0.87
4C3a2b	Frequency of decision-making	How frequently is the worker required to make decisions that affect other people, the financial resources, and/or the image and reputation of the organization?	0.82
4C3a2a	Impact of decisions on co-workers or company results	What results do your decisions usually have on other people or the image or reputation or financial resources of your employer?	0.89
4C3b8	Structured versus unstructured work	To what extent is this job structured for the worker, rather than allowing the worker to determine tasks, priorities, and goals?	0.79
(D) *Machine dependency*			
4A3a3	Controlling machines and processes	Using either control mechanisms or direct physical activity to operate machines or processes (not including computers or vehicles)	0.89
4A3a2	Handling moving objects	Using hands and arms in handling, installing, positioning, and moving materials, and manipulating things	0.89
4A3a4	Operating vehicles, mechanized devices, or equipment	Running, manoeuvring, navigating, or driving vehicles or mechanized equipment, such as forklifts, passenger vehicles, aircraft, or watercraft	0.74
(E) *Time pressure*			
4C3d1	Time pressure	How often does this job require the worker to meet strict deadlines?	1

Obtained from O*NET release 15.1 (February 2011), files “Work Activities” and “Work Context”

**Table 9:** Correlations between different job characteristics (all countries pooled)

	Public	Temporary	Contact	Autonomy	Time pressure	Leadership	Machine-dependent
Public	1						
Temporary	− 0.0434	1					
Contact	0.2185	− 0.0672	1				
Autonomy	0.1344	− 0.1056	0.6808	1			
Time pressure	− 0.0664	− 0.0636	0.0596	0.3679	1		
Leadership	0.2200	− 0.0674	0.4854	0.5195	0.1251	1	
Machine-dependent	− 0.1900	0.0628	− 0.5906	− 0.3386	0.0181	− 0.1712	1

**Table 10:** Prevalence of occupational characteristics among non-parents and parents with a child under 12

	Contact		Autonomy		Time pressure		Leadership		Machine-dependent	
	(1)	(2)	(3)	(4)	(5)	(6)	(7)	(8)	(9)	(10)
	Non-parents	With child under 12 years old	Non-parents	With child under 12 years old	Non-parents	With child under 12 years old	Non-parents	With child under 12 years old	Non-parents	With child under 12 years old
Female	0.25***	0.15***	− 0.00	− 0.16***	− 0.10***	− 0.15***	− 0.19***	− 0.33***	− 0.36***	− 0.35***
	(0.022)	(0.026)	(0.024)	(0.032)	(0.028)	(0.030)	(0.023)	(0.030)	(0.021)	(0.025)
	[***]		[***]		[]		[*]		[***]	
Secondary education	0.28*** (0.031)	0.37*** (0.046)	0.31*** (0.033)	0.34*** (0.044)	0.15*** (0.042)	0.01 (0.047)	0.05 (0.030)	0.08** (0.036)	− 0.27*** (0.030)	− 0.31*** (0.034)
Tertiary education	0.65*** (0.037)	0.78*** (0.047)	0.67*** (0.042)	0.75*** (0.049)	0.22*** (0.049)	0.14*** (0.048)	0.50*** (0.039)	0.61*** (0.048)	− 0.85*** (0.034)	− 0.96*** (0.038)
Public sector	0.08*** (0.030)	0.03 (0.030)	0.13*** (0.036)	0.01 (0.032)	0.10** (0.049)	− 0.03 (0.043)	0.23*** (0.038)	0.14*** (0.044)	0.09** (0.036)	0.11*** (0.030)
Temporary contract	− 0.09*** (0.025)	− 0.19*** (0.027)	− 0.14*** (0.030)	− 0.19*** (0.035)	− 0.09*** (0.032)	− 0.10** (0.042)	− 0.06** (0.023)	− 0.11*** (0.033)	0.08*** (0.025)	0.12*** (0.024)
Constant	− 0.68***	− 0.46	− 0.75***	− 0.67**	0.04	− 0.66*	− 0.64***	− 0.11	− 0.64***	0.43*
	(1.001)	(3.574)	(1.191)	(2.969)	(1.209)	(3.581)	(0.882)	(3.792)	(0.867)	(3.733)
Observations	12,768	11,392	12,768	11,392	12,768	11,392	12,768	11,392	12,768	11,392
R-squared	0.383	0.357	0.244	0.242	0.204	0.200	0.234	0.221	0.452	0.432

Weighted analytic sample. All models include controls for age, age squared, sector at the ISIC 2-digit level, and country fixed effects. Parents defined as responding yes to: “Do you have a child?.” Asterisks within square brackets indicate significance tests conducted in a fully interacted model to test differences between non-parents (here reported) and parents, reported in Table 2

Paired jackknife standard errors in parentheses ****p* < 0.01; ***p* < 0.05; **p* < 0.1

**Table 11:** Prevalence for all workers comparing different standard errors

	Contact			Autonomy			Time pressure			Leadership			Machine-dependent		
	(1)	(2)	(3)	(4)	(5)	(6)	(7)	(8)	(9)	(10)	(11)	(12)	(13)	(14)	(15)
	C	O	C*O	C	O	C*O	C	O	C*O	C	O	C*O	C	O	C*O
Female	0.12***	0.12*	0.12***	− 0.13***	− 0.13**	− 0.13***	− 0.14***	− 0.14**	− 0.14***	− 0.29***	− 0.29***	− 0.29***	− 0.34***	− 0.34***	− 0.34***
	(0.017)	(0.060)	(0.023)	(0.018)	(0.059)	(0.024)	(0.019)	(0.058)	(0.024)	(0.021)	(0.069)	(0.027)	(0.021)	(0.051)	(0.021)
Secondary education	0.36***	0.36***	0.36***	0.36***	0.36***	0.36***	0.10***	0.10***	0.10***	0.16***	0.16**	0.16***	− 0.30***	− 0.30***	− 0.30***
	(0.030)	(0.068)	(0.029)	(0.027)	(0.064)	(0.029)	(0.016)	(0.035)	(0.026)	(0.034)	(0.067)	(0.030)	(0.026)	(0.034)	(0.020)
Tertiary education	0.78***	0.78***	0.78***	0.79***	0.79***	0.79***	0.26***	0.26***	0.26***	0.67***	0.67***	0.67***	− 0.88***	− 0.88***	− 0.88***
	(0.047)	(0.119)	(0.043)	(0.041)	(0.115)	(0.043)	(0.024)	(0.076)	(0.037)	(0.049)	(0.114)	(0.045)	(0.038)	(0.060)	(0.029)
Public sector	0.04*	0.04	0.04	0.03	0.03	0.03	0.01	0.01	0.01	0.14**	0.14***	0.14***	0.04**	0.04	0.04**
	(0.019)	(0.034)	(0.025)	(0.027)	(0.043)	(0.031)	(0.030)	(0.062)	(0.045)	(0.047)	(0.038)	(0.029)	(0.017)	(0.030)	(0.022)
Temporary contract	− 0.12***	− 0.12***	− 0.12***	− 0.17***	− 0.17***	− 0.17***	− 0.08***	− 0.08***	− 0.08***	− 0.10***	− 0.10***	− 0.10***	0.08***	0.08***	0.08***
	(0.015)	(0.024)	(0.018)	(0.024)	(0.027)	(0.020)	(0.022)	(0.025)	(0.021)	(0.017)	(0.023)	(0.017)	(0.012)	(0.019)	(0.013)
Constant	− 0.48***	− 0.48***	− 0.48***	− 0.69***	− 0.69***	− 0.69***	− 0.28**	− 0.28	− 0.28	− 0.42***	− 0.42**	− 0.42***	0.61***	0.61***	0.61***
	(0.085)	(0.144)	(0.129)	(0.077)	(0.159)	(0.128)	(0.098)	(0.215)	(0.174)	(0.122)	(0.164)	(0.145)	(0.076)	(0.129)	(0.109)
Obs	36,464	36,464	36,464	36,464	36,464	36,464	36,464	36,464	36,464	36,464	36,464	36,464	36,464	36,464	36,464
R-squared	0.348	0.348	0.348	0.220	0.220	0.220	0.183	0.183	0.183	0.229	0.229	0.229	0.423	0.423	0.423

Weighted analytic sample. All models include controls for age, age squared, sector at the ISIC 2-digit level, and country fixed effects. This table only contains all workers, results by parenthood status are available upon request. Same models for each factor, only changing the standard error computations. Standard errors clustered by country (C), occupation (O), and country*occupation (C*O)

****p* < 0.01; ***p* < 0.05; **p* < 0.1

**Table 12:** Full wage (including bonuses) models with and without female interactions by parenthood and age of the child

	All workers		Childless only		Parents only		Parents with a child less than 12 years old		Parents with a child less than 18 years old	
	(1)	(2)	(3)	(4)	(5)	(6)	(9)	(10)	(11)	(12)
Female	− 0.15***	− 0.16***	− 0.19**	− 0.19*	− 0.11**	− 0.13**	− 0.15***	− 0.17***	− 0.13***	− 0.16***
	(0.047)	(0.053)	(0.089)	(0.096)	(0.051)	(0.056)	(0.045)	(0.035)	(0.035)	(0.031)
Secondary education	0.14**	0.15**	0.13	0.13	0.16***	0.16***	0.19***	0.19***	0.20***	0.19***
	(0.059)	(0.057)	(0.097)	(0.099)	(0.056)	(0.054)	(0.048)	(0.048)	(0.037)	(0.037)
Tertiary education	0.38***	0.38***	0.26**	0.25**	0.46***	0.47***	0.46***	0.46***	0.48***	0.48***
	(0.082)	(0.082)	(0.115)	(0.119)	(0.076)	(0.078)	(0.058)	(0.061)	(0.052)	(0.055)
Public sector	0.04	0.04	0.14	0.14	0.03	0.04	0.02	0.03	− 0.05	− 0.04
	(0.055)	(0.054)	(0.105)	(0.104)	(0.063)	(0.062)	(0.038)	(0.037)	(0.056)	(0.056)
Temporary contract	− 0.19***	− 0.19***	− 0.20***	− 0.20***	− 0.18***	− 0.18***	− 0.03	− 0.03	− 0.09	− 0.08
	(0.042)	(0.042)	(0.072)	(0.072)	(0.058)	(0.059)	(0.084)	(0.084)	(0.066)	(0.067)
Contact	− 0.10**	− 0.12**	− 0.15*	− 0.16	− 0.07	− 0.10**	− 0.07**	− 0.10**	− 0.09**	− 0.14***
	(0.043)	(0.051)	(0.085)	(0.127)	(0.048)	(0.041)	(0.032)	(0.042)	(0.037)	(0.033)
Female#contact		0.03		0.01		0.04		0.07*		0.08
		(0.092)		(0.155)		(0.090)		(0.040)		(0.064)
Autonomy	0.08**	0.10**	0.11*	0.13*	0.06	0.08*	0.04	0.04	0.05**	0.07
	(0.035)	(0.042)	(0.058)	(0.079)	(0.036)	(0.044)	(0.032)	(0.053)	(0.023)	(0.045)
Female#autonomy		− 0.05		− 0.07		− 0.05		− 0.00		− 0.04
		(0.058)		(0.096)		(0.075)		(0.063)		(0.072)
Time pressure	0.01	− 0.03	− 0.02	− 0.08	0.03	0.03	0.03	0.02	0.03	0.02
	(0.019)	(0.039)	(0.040)	(0.069)	(0.022)	(0.047)	(0.034)	(0.077)	(0.026)	(0.060)
Female#time pressure		0.06		0.13		− 0.00		0.01		0.00
		(0.047)		(0.087)		(0.056)		(0.078)		(0.064)
Leadership	0.10***	0.13***	0.08**	0.10	0.10***	0.14***	0.12***	0.18***	0.11***	0.17***
	(0.023)	(0.039)	(0.042)	(0.068)	(0.030)	(0.045)	(0.030)	(0.057)	(0.029)	(0.044)
Female#leadership		− 0.06		− 0.02		− 0.08		− 0.13*		− 0.11*
		(0.049)		(0.076)		(0.057)		(0.067)		(0.061)
Machine-dependent	− 0.11***	− 0.10***	− 0.18***	− 0.18**	− 0.06**	− 0.05	− 0.07*	− 0.06	− 0.11***	− 0.07**
	(0.031)	(0.036)	(0.065)	(0.084)	(0.030)	(0.032)	(0.035)	(0.051)	(0.032)	(0.034)
Female#machine		− 0.02		0.02		− 0.04		− 0.03		− 0.11**
		(0.048)		(0.106)		(0.050)		(0.053)		(0.051)
Constant	1.75***	1.76***	1.43***	1.46***	1.68***	1.67***	2.35***	2.33***	1.67***	1.64***
	(0.321)	(0.321)	(0.438)	(0.438)	(0.477)	(0.476)	(0.605)	(0.607)	(0.480)	(0.489)
Observations	36,206	36,206	12,667	12,667	23,528	23,528	11,314	11,314	15,273	15,273
R-squared	0.084	0.084	0.082	0.083	0.096	0.096	0.140	0.142	0.113	0.115

Weighted analytic sample. Dependent variable is hourly wages including bonuses. All models include controls for age, age squared, sector at the ISIC 2-digit level, and country fixed effects. Parents defined as responding yes to: “Do you have a child?.” Last four columns include parents who have at least one child under the indicated age

Paired jackknife standard errors in parentheses ****p* < 0.01; ***p* < 0.05; **p* < 0.1

**Table 13:** Wage Models with Female Interaction by Parenthood and Country Groups

	Continental		Eastern		Southern		UK		Denmark	
	(1)	(2)	(3)	(4)	(5)	(6)	(7)	(8)	(9)	(10)
	Non-parents	With child under 12 years old	Non-parents	With child under 12 years old	Non-parents	With child under 12 years old	Non-parents	With child under 12 years old	Non-parents	With child under 12 years old
Female	− 0.33	− 0.15**	− 0.13***	− 0.22***	− 0.04	− 0.14***	− 0.05***	− 0.17***	− 0.04*	− 0.07***
	(0.212)	(0.062)	(0.028)	(0.031)	(0.031)	(0.025)	(0.031)	(0.034)	(0.027)	(0.023)
Secondary education	0.23	0.33***	0.17*	− 0.06	0.13***	0.04	0.21***	0.09	0.28***	0.15***
	(0.193)	(0.115)	(0.100)	(0.125)	(0.033)	(0.027)	(0.077)	(0.071)	(0.054)	(0.049)
Tertiary education	0.20	0.59***	0.36***	0.19	0.28***	0.31***	0.42***	0.32***	0.40***	0.33***
	(0.259)	(0.137)	(0.101)	(0.132)	(0.044)	(0.034)	(0.075)	(0.087)	(0.070)	(0.051)
Public sector	0.17	− 0.00	0.04	0.04	0.08**	0.12***	0.08	0.09*	− 0.13**	− 0.08***
	(0.202)	(0.067)	(0.045)	(0.045)	(0.037)	(0.042)	(0.061)	(0.044)	(0.044)	(0.028)
Temporary contract	− 0.28*	0.16	− 0.10***	− 0.07**	− 0.10***	− 0.10***	− 0.04	− 0.08	− 0.16***	− 0.06**
	(0.162)	(0.229)	(0.026)	(0.030)	(0.029)	(0.026)	(0.046)	(0.053)	(0.042)	(0.029)
Contact	− 0.30	− 0.15	− 0.08***	− 0.12***	0.04	− 0.05	− 0.06	− 0.06	− 0.04	− 0.04
	(0.292)	(0.093)	(0.029)	(0.029)	(0.036)	(0.033)	(0.040)	(0.055)	(0.037)	(0.033)
Female#contact	− 0.02	0.15	0.02	0.01	− 0.06	0.00	0.07	0.06	− 0.02	0.01
	(0.337)	(0.090)	(0.036)	(0.044)	(0.040)	(0.040)	(0.055)	(0.066)	(0.047)	(0.036)
Autonomy	0.21	0.05	0.03	0.05	0.01	0.08**	0.02	0.11**	0.09**	0.08**
	(0.170)	(0.104)	(0.029)	(0.034)	(0.027)	(0.030)	(0.047)	(0.044)	(0.037)	(0.035)
Female#autonomy	− 0.09	− 0.01	0.03	− 0.02	− 0.01	− 0.02	− 0.08	− 0.10	− 0.03	− 0.04
	(0.202)	(0.124)	(0.039)	(0.049)	(0.036)	(0.048)	(0.055)	(0.076)	(0.043)	(0.045)
Time pressure	− 0.17	0.05	0.00	− 0.03*	− 0.02	0.00	0.07**	− 0.00	− 0.03	0.01
	(0.151)	(0.176)	(0.021)	(0.019)	(0.019)	(0.015)	(0.030)	(0.031)	(0.021)	(0.023)
Female#time pressure	0.26	− 0.02	0.00	0.08*	0.04	0.01	− 0.01	0.11***	− 0.01	0.00
	(0.192)	(0.179)	(0.027)	(0.027)	(0.025)	(0.020)	(0.038)	(0.039)	(0.035)	(0.031)
Leadership	0.16	0.27**	0.08***	0.09**	0.05**	0.06***	0.10***	0.07**	0.05	0.04**
	(0.127)	(0.110)	(0.025)	(0.025)	(0.021)	(0.022)	(0.037)	(0.027)	(0.030)	(0.017)
Female#leadership	− 0.08	− 0.25*	0.05	0.06*	0.05**	− 0.01	− 0.02	0.02	− 0.03	0.03
	(0.150)	(0.128)	(0.030)	(0.031)	(0.025)	(0.028)	(0.037)	(0.052)	(0.030)	(0.022)
Machine-dependent	− 0.28	− 0.03	− 0.11***	− 0.14***	− 0.02	− 0.09***	− 0.14***	− 0.10***	− 0.03	− 0.03
	(0.190)	(0.103)	(0.022)	(0.022)	(0.021)	(0.022)	(0.028)	(0.033)	(0.023)	(0.027)
Female#machine	− 0.06	− 0.04	− 0.01	− 0.03	− 0.02	− 0.00	0.13***	− 0.03	− 0.05	− 0.03
	(0.207)	(0.109)	(0.029)	(0.030)	(0.030)	(0.031)	(0.046)	(0.052)	(0.040)	(0.030)
Constant	1.69**	2.28*	1.29**	1.20***	1.09***	1.37***	0.73***	1.18***	1.24***	1.58***
	(0.809)	(1.186)	(0.191)	(0.315)	(0.237)	(0.309)	(0.144)	(0.263)	(0.181)	(0.256)
Observations	3833	3761	4986	2128	2118	1759	1006	915	825	1276
R-squared	0.078	0.080	0.267	0.410	0.305	0.505	0.638	0.512	0.545	0.361

Weighted analytic sample. All models include controls for age, age squared, education, public, temporary, sector at the ISIC 2-digit level, and country fixed effects within groups. Parents defined as responding yes to: “Do you have a child?.” See text for country groups

Paired jackknife standard errors in parentheses ****p* < 0.01; ***p* < 0.05; **p* < 0.1

## Footnotes

1 We explicitly do not consider computers as “machines” in this setting.

2 We use O*NET release 15.1 (February 2011) to reflect the fact that most data come from PIAAC’s first cycle first round collected in 2011–12. The only exceptions are Greece, Lithuania, and Slovenia that participated in PIAAC’s second round in 2014–15.

3 This is defined as the logarithm of gross hourly earnings excluding bonuses for wage and salary earners, PPP corrected $US. Results are robust to using either monthly wages or log hourly wages with and without including bonuses (Appendix Table 12).

4 Standard errors in the main results are based on the sampling mechanism and PIAAC’s technical report best practice (PIAAC Technical report, 2019). In Appendix Table 11, we conducted additional analyses with clustering at the country, occupation, and country and occupation levels as loci where variation in the independent variables may occur (Moulton, 1990). Results support the robustness of our analyses to alternative standard error specifications.

5 Overall, the differences between mothers and fathers compared to gender differences within childless employees are the largest in absolute values for autonomy and leadership and they are significant as displayed in tests in Table 10.

6 We also ran country by country models, which are available upon request.
